# Integrity in and Beyond Contemporary Higher Education: What Does it Mean to University Students?

**DOI:** 10.3389/fpsyg.2016.01094

**Published:** 2016-08-03

**Authors:** Sarah Shi Hui Wong, Stephen Wee Hun Lim, Kathleen M. Quinlan

**Affiliations:** ^1^Department of Psychology, Faculty of Arts and Social Sciences, National University of SingaporeSingapore, Singapore; ^2^Oxford Learning Institute, University of OxfordOxford, UK

**Keywords:** higher education, integrity, personal and social responsibility, qualitative data, survey research

## Abstract

Research has focused on academic integrity in terms of students’ conduct in relation to university rules and procedures, whereas fewer studies examine student integrity more broadly. Of particular interest is whether students in higher education today conceptualize integrity as comprising such broader attributes as personal and social responsibility. We collected and analyzed qualitative responses from 127 students at the National University of Singapore to understand how they define integrity in their lives as students, and how they envisage integrity would be demonstrated in their lives after university. Consistent with the current literature, our data showed that integrity was predominantly taken as “not plagiarizing (in school)/giving appropriate credit when credit is due (in the workplace)”, “not cheating”, and “completing tasks independently”. The survey, though, also revealed further perceptions such as, in a university context, “not manipulating data (e.g., scientific integrity)”, “being honest with others”, “group work commitments”, “conscience/moral ethics/holding true to one’s beliefs”, “being honest with oneself”, “upholding a strong work ethic”, “going against conventions”, and “reporting others”, as well as, in a workplace context, “power and responsibility and its implications”, “professionalism”, and “representing or being loyal to an organization”. The findings suggest that some students see the notion of integrity extending beyond good academic conduct. It is worthwhile to (re)think more broadly what (else) integrity means, discover the gaps in our students’ understanding of integrity, and consider how best we can teach integrity to prepare students for future challenges to integrity and ethical dilemmas.

## Introduction

Creating and maintaining a culture of integrity are integral to effective teaching, learning, and research. “Integrity” is generally understood to encompass “firm adherence to a code of especially moral or artistic values” (Integrity, n.d.), though this study seeks an elaboration that can guide teaching in higher education. Fundamentally, integrity in academic settings is corrupted when students engage in unfair or dishonest practices such as plagiarism, cheating in assessments, and unauthorized collaboration on assignments. Such instances of academic misconduct undermine scholarship and compromise educators’ ability to accurately assess students. Moreover, breaches of academic integrity erode “truth, academic freedom, courage, quality, and the spirit of free intellectual inquiry” (Bertram Gallant, 2008, p. 2) that are cornerstones of learning communities.

Given the importance of integrity to higher education, it is unsurprising that the perception of a “cheating culture” (Callahan, 2004) in academic settings has sparked growing concern and attention (e.g., Hallak and Poisson, 2007; McCabe et al., 2012). For instance, studies have investigated students’ perception of academic integrity in the contexts of plagiarism, collusion, copyright infringement, and data fabrication to develop effective strategies that promote academic integrity (e.g., Kwong et al., 2010).

Besides a commitment to ethical standards, the etymology of integrity denotes wholeness and completeness (Nillsen, 2005). Thus, integrity refers to the larger quality of “wholeness” of character. Yet, with a few notable exceptions in extant discourse (e.g., Stephens et al., 2000; Nillsen, 2005; Saunders and Butts, 2011), there is relatively little research that considers student integrity more broadly beyond its relation to academic misconduct. This paper addresses that gap.

### Integrity in Higher Education: A Broader Notion of Student Integrity

Gaining a deeper and fuller understanding of integrity can support educational efforts to foster holistic student development and cultivate lifelong integrity that extends beyond the classroom in the form of personal and social responsibility. The Association of American Colleges and Universities [AAC&U] (2007), in its report *College Learning for the New Global Century*, championed the development of personal and social responsibility as an essential learning outcome in higher education. This assertion was affirmed in Dey and Associates’s (2008) campus climate survey of 23,000 undergraduate students and 9,000 campus professionals at 23 institutions, in which the authors reported strong consensus amongst the respondents that personal and social responsibility should be a major focus of college education. Indeed, higher education plays a vital role in shaping citizens who will be the leaders of tomorrow (Colby et al., 2003; Colby and Sullivan, 2009; Quinlan, 2011). Thus, its civic obligations not only include guiding students’ intellectual development, but also teaching students to use knowledge to responsibly pursue ends larger than the self.

The Association of American Colleges and Universities’s (n.d.) Core Commitments initiative on *Educating Students for Personal and Social Responsibility* outlined five key dimensions of personal and social responsibility:

(1) Striving for excellence: developing a strong work ethic and consciously doing one’s very best in all aspects of college;
(2) Cultivating personal and academic integrity: recognizing and acting on a sense of honor, ranging from honesty in relationships to principled engagement with a formal academic honor code;
(3) Contributing to a larger community: recognizing and acting on one’s responsibility to the educational community and the wider society, locally, nationally, and globally;
(4) Taking seriously the perspectives of others: recognizing and acting on the obligation to inform one’s own judgment; engaging diverse and competing perspectives as a resource for learning, citizenship, and work;
(5) Developing competence in ethical and moral reasoning: developing ethical and moral reasoning in ways that incorporate the other four responsibilities; using such reasoning in learning and in life.

In line with these dimensions, integrity can be viewed in connection to personal and social responsibility, whereby integrity involves a commitment to ethical responsibilities to the self and others.

### Integrity Beyond Higher Education

As emphasized in the Association of American Colleges and Universities’s (n.d.) Core Commitments initiative, students’ integrity has implications beyond academic contexts. Of particular interest is a study by English et al. (2012), in which both undergraduate and graduate students ranked integrity/honesty as the quality most needed for employment out of 26 workplace qualities. In nurturing the leaders of the future, higher education provides a fertile ground for promoting ethical and moral values that are eventually transferable to the workplace. For instance, principles related to honesty in academic settings such as claiming credit only for work personally completed can be similarly applied in professional settings (Rujoiu and Rujoiu, 2014).

To these ends, this study considers how students perceive integrity to be applicable in workplace contexts. This question is highly relevant assuming that preparing students for life after graduation is a fundamental aim of higher education, and that personal and social responsibility simultaneously call for acting on one’s responsibilities toward a wider community. Here, we investigated how undergraduate and graduate students from the National University of Singapore (NUS) regard integrity to be specifically demonstrated in their academic life, as well as how they envision integrity would be demonstrated in their lives following graduation from university.

## Method

### Participants

One hundred and twenty-seven NUS undergraduate and graduate students in all years of study between the ages of 19 and 38 (*M* = 22.15, *SD* = 2.21) were recruited through email requests (39.94% response rate) by the first author and two research assistants in the Cognition and Education Laboratory at the NUS Department of Psychology. The sample was selected on the basis that respondents were current students at the university. The faculties that the students represented included: Architecture (1%), Arts and Social Sciences (72%), Business (4%), Computing (3%), Dentistry (2%), Engineering (3%), Law (2%), Medicine (2%), and Science (8%). Three percent of respondents declined to state their faculty affiliation.

Participants were informed that the present study aims to define and understand integrity from the student’s perspective, as part of identifying the goal of higher education to help students develop strong personal qualities and character crucial for their effectiveness and success in the future. This research was conducted with the appropriate ethics review board approval by the NUS. Participation in the study was voluntary, and participants were assured anonymity.

### Qualitative Questionnaire

To probe students’ views on integrity, a qualitative online questionnaire consisting of three open-ended items was developed and distributed. Participants first completed the following two sentences as many times as they wished, expressing each idea in a separate phrase:

(1) A specific way that *current university students* demonstrate “Integrity” is…
(2) A specific way that *university graduates* demonstrate “Integrity” is…

Finally, participants were asked to describe any personal experiences through the course of their university education during which they felt they developed “Integrity”.

Participants’ qualitative responses were coded by two independent coders using a process of inductive thematic analysis (Braun and Clarke, 2006). Cohen’s kappa between both coders was 0.92, indicating high inter-coder reliability. Discrepancies (specifically 0.69% of the data) were reviewed and resolved through discussion to produce consensus. Frequency counts are not included here as the aim of the present paper is to represent the widest range of ideas to advance a broader perspective on integrity in relation to university education.

## Results

Responses to the first questionnaire prompt are summarized, followed by responses to the second prompt.

### How Do Students Define Integrity in a University Context?

Consistent with the extant literature, participants predominantly perceived integrity in academic settings as: not plagiarizing, not cheating, and completing tasks independently.

#### Not Plagiarizing

Integrity was defined as “taking effortful steps and measures to avoid plagiarism”, such as “ensuring that work is original and not copied from others” and “acknowledging the sources of [one’s] ideas”.

#### Not Cheating

Participants associated academic integrity with “not cheating” in examinations and assignments, including “not communicating with each other during the final exams”.

#### Completing Tasks Independently

Participants also wrote that completing work such as take-home assignments “on their own without assistance from friends”, “not asking someone else to complete an assignment on their behalf”, and “following instructions on assessments, e.g., no discussing or collaborating” demonstrate integrity in higher education.

Importantly, beyond these themes, participants perceived academic integrity to relate to: not manipulating data, being honest with oneself and others, fulfilling group work commitments, maintaining moral ethics and holding true to one’s beliefs and ideals, upholding a strong work ethic, going against conventions, and reporting ethical violations.

#### Not Manipulating Data

Scientific integrity in the form of “not manipulating data” but “using ethical methods to complete [one]’s experiments, even if it is harder” was raised as an instance of student integrity.

#### Being Honest with Oneself and Others

Participants also suggested that current university students demonstrate integrity by being “truthful and honest about [their] actions and words”, as well as “being open and honest” with themselves and toward others. For instance, this may be displayed through “giving [one’s] honest stances on issues, even when doing so requires more work and persuasion to be done”.

#### Fulfilling Group Work Commitments

Besides completing assignments independently, participants also recognized that fulfilling their group work commitments—such as “contributing equally during group projects”, “ensuring that they do their fair share during projects”, and “accurately reporting their contributions in a group project”—is a demonstration of integrity.

#### Maintaining Moral Ethics and Holding True to One’s Beliefs and Ideals

Participants’ responses revealed that integrity in academic settings is associated with maintaining moral ethics through “not compromising [one’s] conscience”, “not succumbing to peer pressure in circumstances where honesty is easily compromised”, and being consistent with both an “objective moral standard” and “subjective, personal morality in the face of opposition”. In addition, participants opined that integrity is demonstrated by holding true to one’s beliefs and ideals “based on knowledge and instinct”, as illustrated in a personal experience shared by a participant:

In my honors thesis, I stuck to a topic that I felt deeply for. Even though the results were not good, I stuck through it and did not give up. This made me feel like I had integrity toward my ideals and not be a sellout [sic].

#### Upholding a Strong Work Ethic

Integrity was further perceived to be demonstrated through a strong work ethic, as manifested by committing to one’s academic responsibilities. For example, one student noted that:

[…] university education tests integrity constantly—in showing up for classes, or doing the work assigned diligently, even in how one conducts oneself with professors and classmates. One example I suppose is choosing not to compromise in the quality of work submitted—i.e., pushing yourself to do more when you know you can do better.

#### Going against Conventions

The courage to “go against conventions if necessary” and “explore areas that may be sensitive or ‘hard”’ was highlighted as an element of integrity, supporting Kohlberg’s (1973) “post-conventional moral thinking” whereby moral reasoning is based on autonomous judgment and internalized principles, apart from social norms and authorities. As a student described:

When I matriculated, I chose to take modules that I knew I would learn the most from rather than modules that were “easy” and which I had less interest in. I made the pursuit of knowledge rather than grades my end goal, trusting that the grades would follow if I have learnt well.

#### Reporting Ethical Violations

Participants also perceived integrity to include internal whistleblowing, such as “reporting someone who has cheated on an exam”.

### How Do Students Define Integrity in a Workplace Context?

Participants’ definitions of integrity in a workplace context overlapped with those in a university context. Reframed in a workplace setting, these themes included: not plagiarizing (e.g., “giving credit where it is due and not taking credit for work that is not theirs”), being honest with others (e.g., “[describing] personal qualities truthfully during a job interview”), holding true to one’s beliefs and ideals (e.g., “being true to their values at the workplace”), and upholding a strong work ethic (e.g., “adhering to a rigorous work ethic such as being honest in their professional work, not cutting corners” and “not sacrificing honesty and hard work for other methods of career advancement”).

Interestingly, however, participants considered additional concepts when discussing integrity in the workplace, such as: power and responsibility and its implications, professionalism, and representing or being loyal to an organization.

#### Power and Responsibility and Its Implications

Participants perceived integrity in the workplace to be demonstrated through “not misusing their responsibility and power”, “[taking] responsibility of [their] wrongdoings”, and “not shirking responsibilities”. Notably, participants highlighted that university graduates carry the responsibility of harnessing their knowledge, skills, and experiences gained from higher education to “better society in general” as “intellectual frontrunners”. As one student suggested, “graduates who go on to do work such as counselors or lawyers that help the less privileged would be demonstrating integrity”. Besides placing the larger community before the self, university graduates may display integrity by “standing up for what they think is right” and “being unafraid to challenge sociocultural norms in their capacity as leaders and intellectuals to bring about progressive change”.

#### Professionalism

Integrity in the workplace was also defined by students as “exhibiting professionalism in their areas of work”. While there is some ambiguity as to how participants specifically operationalized “professionalism”, it may potentially be related to “adhering to professional ethical principles” and “carrying out fair and just practices in their work life”.

#### Representing or Being Loyal to an Organization

Participants further viewed integrity in the workplace to be demonstrated through “[behaving] in a way that would make NUS [their alma mater] shine” and “performing their jobs by putting the interest of the company before themselves, and aligning their actions with what the company intends to achieve”. Their responses indicated that integrity is associated with actively abiding by the values of an organization with which they are affiliated and representing the organization in a positive light.

## Discussion

Developing students’ integrity is an essential learning outcome in higher education in the US (e.g., the Association of American Colleges and Universities [AAC&U], 2007), and in Singapore where integrity is one of six core values in the 21st Century Competencies framework advanced by the Singapore Ministry of Education (2010). The present study has revealed that university students’ conceptualizations of integrity extend beyond academic misconduct such as plagiarizing and cheating, even though these themes dominate the academic integrity literature (see, e.g., Bertram Gallant, 2008; Kwong et al., 2010). Notably, participants’ responses overlapped with the key dimensions of personal and social responsibility outlined by the Association of American Colleges and Universities (n.d.), including striving for excellence through upholding a strong work ethic, and cultivating personal and academic integrity through being honest with oneself and others. Several of these themes resurfaced in participants’ discussion of how university graduates display integrity, suggesting that notions of academic integrity such as giving credit where it is due are potentially transferrable to workplace contexts. Participants also recognized that using their knowledge and skills responsibly to positively impact society after graduation demonstrates integrity, consistent with the Association of American Colleges and Universities’s (n.d.) emphasis on contributing to a larger community. Interestingly, however, participants further associated integrity with holding true to one’s beliefs and ideals in the face of opposition. This diverges from the Association of American Colleges and Universities’s (n.d.) definition of personal and social responsibility as “taking seriously the perspectives of others” through considering competing perspectives to inform one’s judgments.

### Practical Implications

Critics have noted that the role of higher education in nurturing students’ civic responsibility has often been compromised (Sax, 2000; Kezar, 2004), and that ethics education has not always been successfully integrated in higher education curriculum (Haas, 2005). In business education, for example, content on ethics and social responsibility has been found to be lacking (Nicholson and DeMoss, 2009), even while social responsibility has risen in the corporate agenda (Cornelius et al., 2007). Against such a backdrop, developing students’ holistic moral reasoning beyond good academic conduct assumes particular importance, especially as education and support from mentors may promote students’ development of moral judgments and noble purposes in life (Bronk, 2012; Han and Jeong, 2014). It is thus worthwhile for educators to (re)consider integrity more broadly and guide students to think about integrity more deeply. For instance, although being true to one’s thinking lies at the core of intellectual integrity (Elder and Paul, 1998), the open-mindedness to evaluate competing views, as well as the willingness to revise one’s views where warranted, are also key to critical thinking (Facione, 1990). As educators, we aim to expose students to diverse ideas, challenge their unquestioned assumptions, and encourage them to think analytically in order to develop a mature identity grounded in integrity and rigorous thinking (Colby and Sullivan, 2009). Therefore, to the extent that “holding true” to their beliefs increases students’ resistance to considering opposing perspectives, it may be imperative for educators to guide students to deepen their ethical reasoning.

In addition, students who engage in academic dishonesty are often more likely to continue with such misconduct in the workplace (Rujoiu and Rujoiu, 2014), underscoring the importance of an early start in equipping students to manage ethical dilemmas when their integrity will be tested and, potentially, compromised. For instance, integrity can be embedded into higher education through discussing ethical values and practices in the classroom, role modeling standards of integrity, and building students’ profession-specific ethics expertise via a well-validated ethics curriculum and assessing relevant outcomes (e.g., Bebeau and Thoma, 1998; Löfström et al., 2015).

Educational institutions can also examine how integrity at the individual level can be applied to higher organizational levels in order to develop a multi-level model of integrity (e.g., Palanski and Yammarino, 2007). Through championing organizational virtues that promote structural and ethical integrity based on coherence, consistence, and moral soundness (Young, 2011), universities may be able to nurture integrity in students more effectively. For instance, at the State of the University Address in 2014, NUS President Professor Tan Chorh Chuan unveiled a series of personal qualities including integrity, which the university aims to inculcate in students through new educational initiatives (National University of Singapore Office of the President, 2014). Such institutional commitment to cultivating student integrity is a promising start, particularly since Dey and Associates’s (2008) campus climate survey showed that relatively few undergraduate students and campus professionals perceived their institutions to actually focus on personal and social responsibility, despite the overwhelming agreement that this learning outcome ought to be a major focus of college education.

## Author Contributions

SL and KQ conceptualized the research; all authors developed it. SW collected the data; all authors analyzed them. SW wrote the manuscript with inputs from SL and KQ.

## Conflict of Interest Statement

The authors declare that the research was conducted in the absence of any commercial or financial relationships that could be construed as a potential conflict of interest.
